# Trends in Autism Spectrum Disorder Diagnoses in Japan, 2009 to 2019

**DOI:** 10.1001/jamanetworkopen.2021.9234

**Published:** 2021-05-04

**Authors:** Daimei Sasayama, Rie Kuge, Yuki Toibana, Hideo Honda

**Affiliations:** 1 Department of Psychiatry, Shinshu University School of Medicine, Matsumoto, Nagano, Japan; 2 Department of Child and Adolescent Developmental Psychiatry, Shinshu University School of Medicine, Matsumoto, Nagano, Japan; 3 Mental Health Clinic for Children, Shinshu University Hospital, Matsumoto, Nagano, Japan

## Abstract

This cohort study examines the geographical variations in the incidence of autism spectrum disorder (ASD) and calculates the nationwide cumulative incidence of ASD in Japan.

## Introduction

A recent large-scale birth cohort study in Denmark reported that the diagnosis of autism spectrum disorder (ASD) is increasing and that its future cumulative incidence could exceed 2.8%.^1^ In Japan, 3 recent cohort studies^2,3,4^ have consistently reported prevalence or incidence of ASD exceeding 3%. The question may arise: do these relatively high figures compared with worldwide data (eTable in the Supplement) represent the nationwide incidence in Japan? To answer this question, we analyzed the data from the National Database (NDB) of Health Insurance Claims of Japan.^5^ We examined the geographical variations in the incidence of ASD and calculated the nationwide cumulative incidence of ASD in Japan.

## Methods

This cohort study was approved by the ethics committee of Shinshu University School of Medicine. Informed consent was not required because of the anonymous nature of the data. This study was reported following the Strengthening the Reporting of Observational Studies in Epidemiology (STROBE) reporting guideline. No statistical tests were conducted in this study. A detailed description of the methods is shown in the eAppendix in the Supplement. Data on children born in fiscal years (April 1 to March 31) 2009 to 2016 and diagnosed with ASD (*International Statistical Classification of Diseases, Tenth Revision *[*ICD-10*] code F84) in fiscal years 2009 to 2019 were retrieved from the NDB. Extracted information included sex, the year and age at diagnosis, and the prefecture where ASD was diagnosed. Data analysis was conducted between December 2020 and March 2021.

Because the annual number of births in each fiscal year was not available, the number of births in each calendar year was used to estimate the lifetime cumulative incidence by dividing the number of children diagnosed in a fiscal year by the number of births for that calendar year. The difference in birth numbers between fiscal years and calendar years is likely to be less than 1% (eAppendix in the Supplement).

## Results

**Figure 1.**
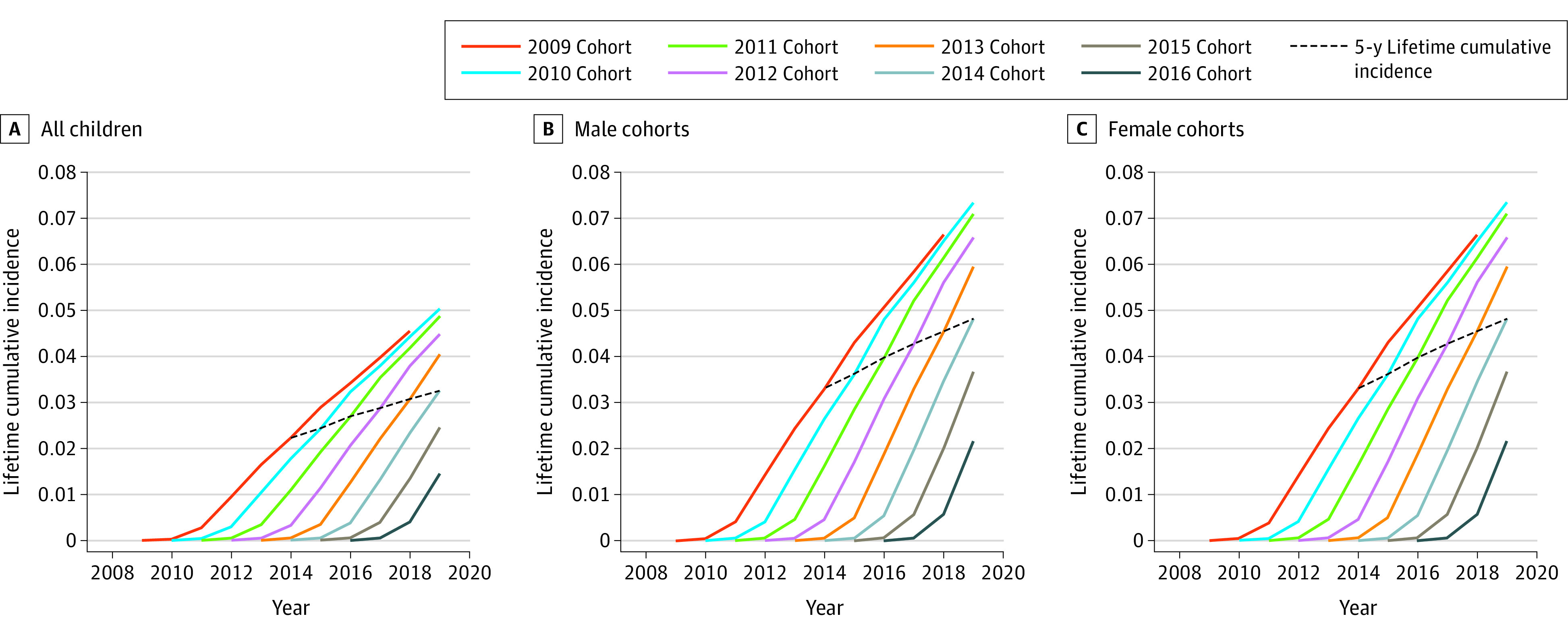
Cumulative Incidence of Autism Spectrum Disorder in Japan Among Children Born in Fiscal Years 2009 to 2016 The lifetime cumulative incidence values each year for all children (A), boys (B), and girls (C) were rounded to the nearest fourth decimal place and plotted. Each curve shows the lifetime cumulative incidence of ASD in children born in each fiscal year. The 5-year lifetime cumulative incidence of ASD increased during the study years.

**Figure 2.**
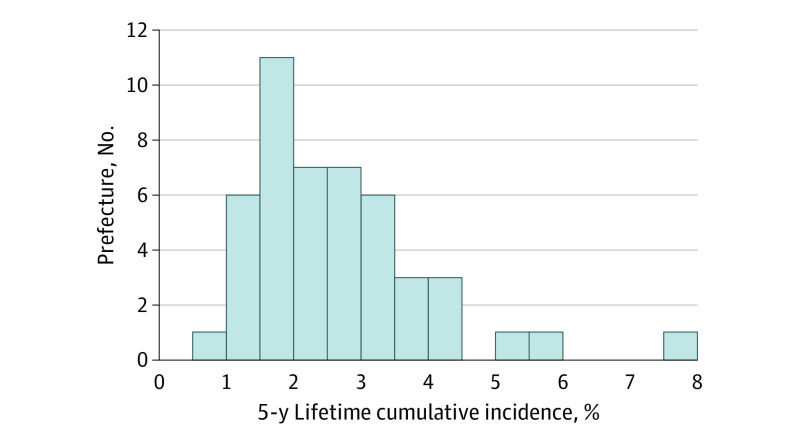
Histogram of 5-Year Lifetime Cumulative Incidence of Autism Spectrum Disorder in Each Prefecture in Children Born in Fiscal Years 2009 to 2014

We extracted data on 313 353 children (236 386 [75.4%] boys and 76 967 [24.6%] girls) born in fiscal years 2009 to 2016 and diagnosed with ASD in fiscal years 2009 to 2019. The number of children born in Japan between 2009 and 2014 was 6 262 731, of whom 172 276 (131 117 [76.1%] boys and 41 159 [23.9%] girls) were diagnosed with ASD in or before the fiscal year 5 years after their birth year. Therefore, the estimated nationwide 5-year lifetime cumulative incidence of ASD in children born between fiscal 2009 and 2014 was 2.75%. The lifetime cumulative incidence for each birth year cohort (Figure 1) showed a steady increase, from 2.23% for the 2009 cohort to 3.26% for the 2014 cohort. Diagnostic incidence per year increased substantially from 2 years after birth and decreased slightly after 6 years after birth in both boys and girls. For example, for the 2009 to 2011 cohorts, the mean incidence within 2 years after birth was 0.0014% per year for boys and 0.0006% per year for girls; the mean incidence from 2 to 6 years after birth was 0.0109% per year for boys and 0.0035% per year for girls; the mean incidence from 7 to 8 years after birth was 0.0086% per year for boys and 0.0033% per year for girls. Figure 2 shows the 5-year lifetime cumulative incidence of ASD in each of the 47 prefectures in Japan. The 5-year lifetime cumulative incidence in each prefecture ranged from 0.9% to 7.9%, with a median of 2.4%. The 5-year lifetime cumulative incidence exceeded 2.0% in 29 prefectures (62%).

## Discussion

This study found that the nationwide cumulative incidence of ASD was comparable with what has been reported in local cohorts^2,4^ and that the diagnosis of ASD increased in Japan between 2009 and 2019. Expanding public awareness may have contributed to the increased nationwide incidence, whereas the regional variation may be because of other etiological and nonetiological (eg, accessibility to services) factors. Our findings indicate an important need for further health services and etiologic research.

Several limitations must be noted. First, our calculations of cumulative incidence did not assume migration. Second, Rett syndrome was counted as ASD because of the use of *ICD-10* code F84. However, the effect of these limitations on the nationwide cumulative incidence is likely to be negligible due to the low rate of migration abroad and the low incidence of Rett syndrome.^6^ In contrast, migration between prefectures may have caused some inaccuracy in the cumulative incidence for each prefecture. Furthermore, the number of births per calendar year was used for analyses, most likely leading to a minor underestimation of the cumulative incidence.

Despite the limitations, this study found that the incidence of ASD in Japan was higher than what has been reported worldwide. The results bring attention to the necessity of developing support systems to meet the needs of an increasing number of individuals diagnosed with ASD.

## Notes

Supplement. **eAppendix.** Supplementary Methods **eTable.** Previously Reported Prevalence and Incidence of Autism Spectrum Disorder **eReferences.**
