# Error in Figure 1

**DOI:** 10.1001/jamanetworkopen.2021.18052

**Published:** 2021-06-14

In the Research Letter titled “Trends in Autism Spectrum Disorder Diagnoses in Japan, 2009 to 2019,”^1^ published May 4, 2021, there was an error in Figure 1. A duplicate of Figure 1B was presented as Figure 1C. This article has been corrected.^1^
