# Supplementary material for: Trends in Autism Spectrum Disorder Diagnoses in Japan, 2009 to 2019
Source: JAMA Netw Open. 2021 May 4;4(5):e219234. doi: 10.1001/jamanetworkopen.2021.9234 (PMC8097493; doi:10.1001/jamanetworkopen.2021.9234)
Supplement: Supplement. — eAppendix. Supplementary Methods eTable. Previously Reported Prevalence and Incidence of Autism Spectrum Disorder eReferences. [file jamanetwopen-e219234-s001.pdf]

Supplemental Online Content

Sasayama D, Kuge R, Toibana Y, Honda H. Trends in autism spectrum disorder diagnoses in Japan, 2009 to 2019. *JAMA Netw Open.* 2021;4(5):e219234. doi:10.1001/jamanetworkopen.2021.9234

eAppendix. Supplementary Methods

eTable. Previously Reported Prevalence and Incidence of Autism Spectrum Disorder

eReferences.

This supplemental material has been provided by the authors to give readers additional information about their work.

© 2021 Sasayama D et al. *JAMA Network Open.*

eAppendix. Supplementary Methods

A retrospective cohort study was conducted using data from the national database of health insurance claims of Japan (NDB). The reporting of this study conforms to the Strengthening the Reporting of Observational Studies in Epidemiology (STROBE) statement for reporting of cohort studies. The NDB includes all electronic-based health insurance claims in Japan since fiscal year 2009. The NDB includes the health insurance claims data such as the patient identification number, sex, age or age group, diagnostic codes, institution identification number, prefecture where the institution is located, procedural codes, and drug codes. Medical treatments completely covered by public funds and those not covered by public insurance are not included. A detailed description of NDB is described in the website of the Ministry of Health, Labour and Welfare.[1] Several retrospective, population-based studies have previously been conducted using the medical records retrieved from the NDB.[2-4]

All permanent residents in Japan are mandatorily enrolled in the national universal health insurance program. Patients are free to choose any clinics or hospitals, and the health insurance covers 70-90% of their medical costs. For children under 16, the rest of the medical cost is generally further subsidized almost completely by municipalities. Therefore, the majority of medical care for children in Japan are likely to be covered by the national healthcare system and thus are included in the NDB.

Data on children born in fiscal years 2009-2016 and diagnosed with ASD (ICD-10 code: F84) in fiscal years 2009-2019 were retrieved from the NDB. Rett's syndrome, coded as F84.2, is not automatically classified as ASD. However, a substantial proportion of patients with Rett's syndrome is likely to have comorbid ASD.[5] Therefore, we identified ASD by the single F84 code, as in some previous studies.[6,7] Furthermore, previous studies reported that the prevalence of Rett's syndrome was less than 0.01%.[8,9] Therefore, it is unlikely that including Rett's syndrome influenced the results of the present study.

Extracted information were sex, the year and age at diagnosis, and the prefecture where ASD was diagnosed. The age at diagnosis was available for only children below 10 years old. For those aged 10 years or above, only the total number of diagnosis for each age group (grouped in 10-year age groups) was retrievable. Therefore, the number of ASD diagnosed in fiscal year 2019 in children born in fiscal year 2009 was not available. The study was approved by the Ethics Committee of Shinshu University School of Medicine. Informed consent was not required due to the anonymous nature of the data.

The annual number of births in Japan in each fiscal year was not available. Therefore, the cumulative incidence of ASD was calculated using the annual number of births in each calendar year, which was retrieved from the data released by the statistics bureau of Japan.[10] The estimated nationwide lifetime cumulative incidence in children born in a certain fiscal year was calculated by the following equation:
(Estimated lifetime cumulative incidence for children born in Japan in fiscal year X) = (Number of diagnosed children born in Japan in fiscal year X)/(Annual number of births in Japan in calendar year X)
The percent change per year in birth numbers in 2009-2014 ranged from -2.6% to +0.1%. Therefore, the difference in birth numbers between the calendar year and the fiscal year, which starts on April 1, is likely to be no more than 1%. Thus, using calendar year, which starts 3 months earlier than the fiscal year, instead of fiscal year to calculate the 5-year lifetime cumulative incidence of ASD in children born in fiscal 2009-2014 was likely to have little influence on the results, most likely a minor underestimation.

The cumulative incidence in each prefecture was calculated similarly using the following equation:
(Estimated lifetime cumulative incidence for children born in prefecture Y in fiscal year X) = (Number of

© 2021 Sasayama D et al. *JAMA Network Open.*

children born in fiscal year X who were diagnosed in prefecture Y)/(Annual number of births in prefecture Y in calendar year X)

Japan is a unitary state divided into 47 prefectures. The basic framework of healthcare in Japan is set tightly by national laws. Therefore, no geographical difference exists with respect to healthcare system. In contrast, a large discrepancy in population and population density exists between prefectures. As of 2014, the population and population density in each prefecture range from 574,022 to 1,3378,584 and from 68.9 to 6106.4/ km^2, respectively, and the annual number of births in 2014 for each prefecture range from 4,527 to 110,629. Therefore, it is likely that life environment and accessibility to services differ substantially throughout Japan.

© 2021 Sasayama D et al. *JAMA Network Open.*

eTable 1. Previously Reported Prevalence and Incidence of Autism Spectrum Disorder					
**Study**	**Time of data collection**	**Denominator (Population size)**	**Frequency**	**Measure of frequency**	**Country**
Nishimura et al[1]	2010-2014	952	3.1%	Cumulative incidence at age 32 months	Japan
Saito et al[2]	2013-2016	5,016	3.22%	Prevalence in children aged 5 years	Japan
Sasayama et al[3]	2016-2018	1,067	3.1%	Cumulative incidence at age 6 years	Japan
Zhou et al[4]	2014-2016	125,806	0.70%	Prevalence in children aged 6-12 years	China
Arora et al[5]	2011-2012	3,964	1.11%	Prevalence in children aged 2-9 years	India
Kim et al[6]	2005-2006	55,266	2.20%	Prevalence in children aged 7-12 years	South Korea
Maenner et al[7]	2016	275,419	1.85%	Prevalence in children aged 8 years	United States
Xu et al[8]	2014-2016	30,502	2.47%	Prevalence in children aged 3 to 17 years	United States
Schendel et al[9]	2016	2,055,928	2.80%	Cumulative incidence in birth cohorts 1980-2012	Denmark
Al-Mamri et al[10]	2011-2018	837,655	0.20%	Prevalence in children aged 0-14 years	Oman
van Bakel et al[11]	2005-2010	307,751	0.365%	Prevalence in children aged 7 years	France
Skonieczna-Żydecka et al[12]	2010-2014	707,975	0.35%	Prevalence in children aged 0-16 years	Poland
Idring et al[13]	2011	735,096	1.54%	Prevalence in 0- to 27-year-olds	Finland

Recent studies reporting prevalence or incidence of autism spectrum disorder in the general population are listed in the table. The regional variations suggest difference in etiologic factors (e.g., environmental factors, genetic factors, and parental age) and non-etiologic factors (e.g., public awareness and accessibility to services) among countries. Recent epidemiological studies in Japan show relatively high prevalence or incidence of autism spectrum disorder compared to that of other countries.

© 2021 Sasayama D et al. *JAMA Network Open.*

References cited in eTable 1

1. Nishimura T, Takei N, Tsuchiya KJ. Neurodevelopmental Trajectory During Infancy and Diagnosis of Autism Spectrum Disorder as an Outcome at 32 Months of Age. Epidemiology. 2019;30 Suppl 1:S9-S14.
2. Saito M, Hirota T, Sakamoto Y, et al. Prevalence and cumulative incidence of autism spectrum disorders and the patterns of co-occurring neurodevelopmental disorders in a total population sample of 5-year-old children. Mol Autism. 2020;11(1):35.
3. Sasayama D, Kudo T, Kaneko W, et al. Brief Report: Cumulative Incidence of Autism Spectrum Disorder Before School Entry in a Thoroughly Screened Population. J Autism Dev Disord. 2020.
4. Zhou H, Xu X, Yan W, et al. Prevalence of Autism Spectrum Disorder in China: A Nationwide Multi-center Population-based Study Among Children Aged 6 to 12 Years. Neurosci Bull. 2020;36(9):961-971.
5. Arora NK, Nair MKC, Gulati S, et al. Neurodevelopmental disorders in children aged 2-9 years: Population-based burden estimates across five regions in India. PLoS Med. 2018;15(7):e1002615.
6. Kim YS, Fombonne E, Koh YJ, Kim SJ, Cheon KA, Leventhal BL. A comparison of DSM-IV pervasive developmental disorder and DSM-5 autism spectrum disorder prevalence in an epidemiologic sample. J Am Acad Child Adolesc Psychiatry. 2014;53(5):500-508.
7. Maenner MJ, Shaw KA, Baio J, et al. Prevalence of Autism Spectrum Disorder Among Children Aged 8 Years - Autism and Developmental Disabilities Monitoring Network, 11 Sites, United States, 2016. MMWR Surveill Summ. 2020;69(4):1-12.
8. Xu G, Strathearn L, Liu B, Bao W. Prevalence of Autism Spectrum Disorder Among US Children and Adolescents, 2014-2016. JAMA. 2018;319(1):81-82.
9. Schendel DE, Thorsteinsson E. Cumulative Incidence of Autism Into Adulthood for Birth Cohorts in Denmark, 1980-2012. JAMA. 2018;320(17):1811-1813.
10. Al-Mamri W, Idris AB, Dakak S, et al. Revisiting the Prevalence of Autism Spectrum Disorder among Omani Children: A multicentre study. Sultan Qaboos Univ Med J. 2019;19(4):e305-e309.
11. van Bakel MM, Delobel-Ayoub M, Cans C, et al. Low but increasing prevalence of autism spectrum disorders in a French area from register-based data. J Autism Dev Disord. 2015;45(10):3255-3261.
12. Skonieczna-Zydecka K, Gorzkowska I, Pierzak-Sominka J, Adler G. The Prevalence of Autism Spectrum Disorders in West Pomeranian and Pomeranian Regions of Poland. J Appl Res Intellect Disabil. 2017;30(2):283-289.
13. Idring S, Lundberg M, Sturm H, et al. Changes in prevalence of autism spectrum disorders in 2001-2011: findings from the Stockholm youth cohort. J Autism Dev Disord. 2015;45(6):1766-1773.

© 2021 Sasayama D et al. *JAMA Network Open.*

eReferences

1. Ministry of health labor and welfare Japan. On the use of the National Database of Health Insurance Claim Information and Specified Medical Checkups (in Japanese). 2013; http://www.mhlw.go.jp/stf/shingi/2r9852000002ss9z-att/2r9852000002ssfg.pdf.
2. Nojiri S, Itoh H, Kasai T, et al. Comorbidity status in hospitalized elderly in Japan: Analysis from National Database of Health Insurance Claims and Specific Health Checkups. *Sci Rep.* 2019;9(1):20237.
3. Ono A, Ishikane M, Kusama Y, et al. The first national survey of antimicrobial use among dentists in Japan from 2015 to 2017 based on the national database of health insurance claims and specific health checkups of Japan. *PLoS One.* 2020;15(12):e0244521.
4. Okumura Y, Sakata N, Takahashi K, Nishi D, Tachimori H. Epidemiology of overdose episodes from the period prior to hospitalization for drug poisoning until discharge in Japan: An exploratory descriptive study using a nationwide claims database. *J Epidemiol.* 2017;27(8):373-380.
5. Wulffaert J, Van Berckelaer-Onnes IA, Scholte EM. Autistic disorder symptoms in Rett syndrome. *Autism.* 2009;13(6):567-581.
6. Hong M, Lee SY, Han J, et al. Prescription Trends of Psychotropics in Children and Adolescents with Autism Based on Nationwide Health Insurance Data. *J Korean Med Sci.* 2017;32(10):1687-1693.
7. Diallo FB, Fombonne E, Kisely S, et al. Prevalence and Correlates of Autism Spectrum Disorders in Quebec: Prevalence et correlats des troubles du spectre de l'autisme au Quebec. *Can J Psychiatry.* 2018;63(4):231-239.
8. Laurvick CL, de Klerk N, Bower C, et al. Rett syndrome in Australia: a review of the epidemiology. *J Pediatr.* 2006;148(3):347-352.
9. Wong VC, Li SY. Rett syndrome: prevalence among Chinese and a comparison of MECP2 mutations of classic Rett syndrome with other neurodevelopmental disorders. *J Child Neurol.* 2007;22(12):1397-1400.
10. Statistics Bureau Ministry of Internal Affairs and Communications. Statistics Bureau of Japan. https://www.stat.go.jp/index.html. Accessed March 9, 2021.

© 2021 Sasayama D et al. *JAMA Network Open.*
